# Advances in ATM, ATR, WEE1, and CHK1/2 inhibitors in the treatment of PARP inhibitor-resistant ovarian cancer

**DOI:** 10.20892/j.issn.2095-3941.2023.0260

**Published:** 2024-02-05

**Authors:** Qin Tang, Xin Wang, Haixia Wang, Lin Zhong, Dongling Zou

**Affiliations:** 1 Department of Gynecologic Oncology, Chongqing University Cancer Hospital & Chongqing Cancer Institute & Chongqing Cancer Hospital, Chongqing 400030, China; 2 Chongqing Specialized Medical Research Center of Ovarian Cancer, Chongqing 400030, China; 3 Organoid Transformational Research Center, Chongqing Key Laboratory of Translational Research for Cancer Metastasis and Individualized Treatment, Chongqing University Cancer Hospital, Chongqing 400030, China

Ovarian cancer (OC) poses a significant challenge in modern gynecologic oncology, both diagnostically and therapeutically. According to the American Cancer Society, an estimated 21,000 new cases of OC were reported in the United States alone in 2021^1^. The most prevalent subtype of OC87, high-grade serous (HGS), is characterized by heightened genomic instability and defects in DNA damage response (DDR) pathways, which contribute to disease development and progression^2^. Notably, approximately 50% of HGS ovarian cancer (HGSOC) patients exhibit homologous recombination repair (HRR) defects (HRDs)^3^.

Targeting DDR defects as a promising strategy in cancer therapy is evidenced by the success of poly-ADP ribose polymerase (PARP) inhibitors (PARPis) in cancers with HRDs^4^. The introduction of PARPi therapy for platinum-sensitive HRD HGSOC has yielded significant clinical benefits, most notable of which is a prolongation of progression-free survival (PFS)^3^. However, the subset of patients with biomarker-negative HRD, which represents approximately one-half of all HGSOCs, experiences minimal benefits, often showing short-lived or no response to maintenance single-agent PARPi therapy. Moreover, PARPi resistance is common in responsive patients^5^. Hence, there is a need to identify new targets and assess novel combinations of targeted therapies in this domain.

The DDR pathway orchestrates cell cycle progression with DNA repair to minimize the passage of DNA damage to daughter cells^6^. Key proteins in signaling DNA damage to cell cycle checkpoints and DNA repair pathways include ataxia-telangiectasia mutated (ATM) kinase, ATM- and Rad3-related (ATR) kinases, and the DNA-dependent protein kinase catalytic subunit (DNA-PKcs). Disruption or overwhelming of these response pathways can result in irreparable damage and cellular death. This vulnerability has been harnessed in the development of PARPis for tumors with defective HRRs. With an enhanced understanding of DNA damage and repair biology, novel DDR-targeting molecules exploiting replication stress through DDR inhibition are emerging as promising anti-cancer therapies^7^.

The stability of replication forks is intricately linked to the cell cycle, with cell cycle checkpoints facilitating the detection of DNA damage signals, activation of signaling pathways, and cell cycle arrest. ATR, CHK1/2, and cyclin-dependent kinase (CDK) have pivotal roles in this process. Notably, Haynes et al.^8^ elucidated the role of cell cycle checkpoint proteins (ATR/CHK1 and WEE1) in stabilizing replication forks, suggesting that combining PARPi therapy with cell cycle checkpoint inhibitors can mitigate drug resistance.

## ATR inhibitor

ATR, a 2660-amino-acid-long mammalian homologue of mitotic entry checkpoint proteins in yeast, belongs to the phosphatidylinositol 3-kinase (PIKK) family^9^. Activated ATR kinase regulates cellular processes, including cell cycle arrest, replication initiation inhibition, deoxynucleotide synthesis promotion, replication fork initiation, and DNA double-strand break (DSB) repair. ATR kinase inhibitors have demonstrated significant potential in killing tumor cells as monotherapy or in combination with other targeted agents.

ATR kinase inhibitors are small molecule inhibitors that target ATR kinase and respond to replication stress (RS) by phosphorylation of CHK1, which triggers cell cycle arrest at S, G2, and M stages and inhibits ATR kinase-mediated repair pathways and efficiently kills tumor cells. ATR kinase inhibitors have shown high potential in killing tumor cells when used as monotherapy or in combination with other targeted agents^10^.

OC inevitably develops platinum and PARPi resistance. lt has been shown that acquired PARPi resistance is often accompanied by increased ATR-CHK1 activity and sensitivity to ATR inhibitors. This finding suggests that PARPi-ATRi may be a promising strategy for PARPi- and platinum-resistant OC independent of the underlying mechanism of resistance^3^. PARPis block single-strand break (SSB) repair, which causes RS, replication fork collapse, and ATR activation. Therefore, the mechanism by which inhibitors combine these two key DDR components involves PARPi-induced RS, which requires transmission of ATR signaling to cell cycle checkpoints to resolve damage. Multiple preclinical studies have shown convincing synergy between ATRis and PARPis in many cancer types, although the putative mechanisms are not entirely consistent between studies. At present, most clinical studies combine an ATRi and PARPi to treat OC. CAPRI (NCT03462342) is an investigator-initiated phase II clinical study of ceralasertib in combination with olaparib in the treatment of recurrent HGS adenocarcinoma^11^. The results to date show that olaparib in combination with ceralasertib is well-tolerated. Although favorable effects have been observed in patients with BRCA1 mutations, there is no objective response^11^.

Early clinical trials have shown the potential activity of various ATRis. The results of a phase I trial indicated that BAY 1895344 at a dose > 40 mg twice daily was well-tolerated and showed anti-tumor activity in 21 patients with heavily pre-treated advanced solid tumors with an objective response rate (ORR) of 30.7% (4/13). All responders had ATM loss or an ATM mutation and one patient with BRCA1m HGSOC who had olaparib- and chemotherapy-resistant disease had a durable response. Elimusertib also showed clinical activity in patients with advanced solid tumors and DDR defects resistant to standard treatment, of whom 45 had gynecologic tumors. A durable clinical benefit lasting > 6 months in 27.8% of patients with OC was achieved, including patients who were resistant to platinum-based chemotherapy and patients with prior PARPi treatment; however, significant grade 3–4 hematologic toxicity was reported^12^. In the phase I TRESR trial, in which RP-3500 was evaluated, 20 patients with DDR pathway-deficient OC were included, 18 progressed on prior PARPi treatment and 17 had platinum-resistant disease. The ORR was 25% and the median PFS was 35 weeks. The median PFS of 2 patients with post-PARPi BRCA1 was 35 weeks. Two patients had post-PARPi BRCA1 reversion mutations following a 17- or 29-week treatment duration.

The combination of an ATRi and PARPi has shown synergy in preclinical studies involving various cancer types, which prompted ongoing clinical trials. The CAPRI trial evaluated the combination of olaparib and ceralasertib in platinum-sensitive HR-deficient HGSOC that progressed during PARPi treatment, which showed clinical benefit > 12 months for first-line and > 6 months for second-line medications. The ORR was 46% and the median PFS was 7.5 months. The olaparib and ceralasertib combination had encouraging clinical activity and acceptable toxicity^13^. The olaparib and ceralasertib combination was also assessed in the OLAPCO trial^14^, which reported an ORR of 14% and a clinical benefit of 86% in 7 patients with PARPi-resistant BRCAm HGSOC. Berzosertib with gemcitabine showed clinical activity in platinum-resistant OC. Of the 70 patients included, 19% and 32% had prior PARPi therapy in the combination and single agent groups, respectively. The median PFS was 22.9 weeks in the combination group and 14.7 weeks in the gemcitabine group (HR 0.57; *P* = 0.04). Interestingly, the RS level was analysed according to a pre-established score (high if at least one RS alteration was present versus low if no RS alterations were present); only patients with a low RS had a PFS benefit by adding berzosertib to gemcitabine, suggesting the potential role of RS as a biomarker and warranting future evaluation.

BRCA reversion mutations and the ability to form RAD51 foci are infrequently observed in models of acquired PARPi resistance, suggesting the existence of alternative resistance mechanisms. However, regardless of the mechanism underlying resistance, complete and durable therapeutic responses to PARPi-ATRi that significantly increase survival have been reported in clinically relevant platinum and acquired PARPi-resistant patient-derived xenograft (PDX) models. These findings indicate that PARPi-ATRi treatment is a highly promising strategy for OCs that acquire resistance to PARPi and platinum. Targeting the RS response is a valid therapeutic option to overcome PARPi resistance, including tumors without an underlying HRR deficiency.

## WEE1 inhibitor

Because most human tumor cells have G1/S phase checkpoint inactivation due to p53 mutations and p53 function defects, human tumor cells are more dependent on G2/M checkpoint regulation. WEE1 kinase, a promising target for cancer therapy, is crucial for S and G2 phase cell cycle checkpoint arrest. WEE1 inhibits CDK1 activity, preventing DNA-damaged cells from entering mitosis. G1/S phase checkpoint inactivation is common in patient with OC due to p53 mutations. WEE1 inhibition can further inactivate the G2/M checkpoint, inducing irreparable DNA damage and apoptosis. Corollary studies have shown that because > 95% of OC patients carry the mutated p53 gene, the G1/S checkpoint is inactivated and WEE1 inhibition can further inactivate the G2/M checkpoint, resulting in irreparable DNA damage and eventual apoptosis of OC cells. WEE1 inhibits the expression of DNA methyltransferase 1 (DNMT1) by downregulating the transcription factor, E2F, signaling pathway, then upregulates endogenous retroviruses, promotes the secretion of interferon by tumor cells, and upregulates the interferon signaling pathway. Programmed death-ligand 1 (PD-L1) is upregulated, which ultimately promotes tumor proliferation. Although WEE1 kinase activity has a crucial role in the signal transduction of tumor cells, thus far most of the reported WEE1 kinase inhibitors have not been used in the clinical setting. The inhibitors that are used in the clinical setting, such as adavosertib, also cause safety problems due to low selectivity and off-target effects. WEE1 inhibitors have been used in combination with other anti-cancer drugs in clinical trials; however, further studies are needed to determine the potential off-target effects and efficacy of these drugs in clinical practice. How to maintain the efficacy of the inhibitor and simultaneously control the balance between WEE1 kinase inhibitors and other drugs or treatments to deliver maximum benefits to patients is a current problem that needs to be solved.

Despite challenges in the clinical development of WEE1 kinase inhibitors, early trials, such as the phase II EFFORT, have shown clinical efficacy in combination with olaparib in PARPi-resistant OC. The preliminary results of the phase II EFFORT showed that as a single agent and in combination with olaparib in OC patients following PARPi progression (a previous benefit from a PARPi was not required), adavosertib yielded an ORR and median PFS of 23% and 5.5 months and 29% and 6.8 months, respectively, in 70 evaluable patients. Grade 3 and 4 toxicities were observed in both groups but were manageable; however, 56% of the patients in the combination arm required dose reduction, and 85% had a dose interruption. Modulating cell cycle checkpoint signaling in OC patients with agents targeting DDR pathways is an exciting option with a mechanistic rationale for combination with PARPi or cytotoxic chemotherapy, as agents targeting DDR pathways have the potential to induce synthetic lethality. All these combinations require comprehensive preclinical data to assess the timing of dosing for the optimal strategy and to limit the induced hematologic toxicities.

Adavosertib is a WEE1 inhibitor that is used in combination with gemcitabine for the treatment of platinum-resistant or -refractory recurrent OC. In a double-blind, randomized, placebo-controlled phase II trial (NCT02151292), the clinical efficacy of a WEE1 inhibitor in combination with gemcitabine was demonstrated, supporting the ongoing evaluation of DDRs in HGS carcinoma, a tumor type with a TP53 mutation and high RS. This therapeutic approach may be applicable to other tumor types with high replication effects; however, larger studies are needed to verify this conclusion^15^. The observed clinical efficacy of a WEE1 inhibitor combined with gemcitabine supports ongoing assessment of DDR drugs in HGSOC, a TP53-mutated tumor type with high RS. This therapeutic approach might be applicable to other tumor types with high RS; larger confirmatory studies are required^15^.

## ATM inhibitor

ATM is a protein kinase with a key role in the DNA DSB repair response^16^. In addition to ATR and DNA-dependent protein kinase (DNA-PK), ATM is part of the PI3K family and imparts essential functions in DDR. When activated by the MRN complex (MRE11-RAD51-NBS1), ATM phosphorylates the H2AX histone at Ser139 (γH2AX), which then directly binds to DNA damage checkpoint protein regulator 1 (MDC1). By amplifying the DNA damage signal, the γH2AX region is further expanded to provide a reaction platform for the recruitment and function of 53BP1. In addition, ATM prevents the MDM2-dependent ubiquitination of p53 proteasome degradation by phosphorylation of p53. *In vitro* cell experiments demonstrated that ATM inhibitors enhance the tumor killing effect of DSB inducers or ionizing radiation. ATM is a recognized tumor suppressor gene. Because the early stage of tumorigenesis is often accompanied by RS or telomere shortening and other processes to activate the DDR pathway, cycle stagnation caused by ARM activation may become a key factor in tumorigenesis and development. During treatment it was shown that patients with ATM mutations have poor sensitivity to radiotherapy and chemotherapy. ATM inhibitors enhance the tumor-killing effect of DNA DSB inducers or ionizing radiation *in vitro* hold promise as sensitizers for radiotherapy.

PHI-101, a CHK2 inhibitor, has demonstrated therapeutic efficacy against OC, especially in cases resistant to existing anti-neoplastic drugs. When HGSOC cell line and various OC cell lines (CAOV3, OVCAR3, SK-OV-03, and SW626) were treated with PHI-101 in a non-clinical study, the therapeutic effect of PHI-101 against ovarian cancer was demonstrated by a decrease in OC cell viability. In addition, a stronger growth inhibition effect was observed compared to treatment with olaparib or rucaparib alone, and a much stronger inhibition effect was observed when concomitantly used with paclitaxel, cisplatin, and topotecan. Based on the aforementioned results of the non-clinical studies, the potential of PHI-101 as a new treatment or concomitant cytotoxic chemotherapeutic for patients with OC who are resistant to existing anti-neoplastic drugs was confirmed^17^.

## CHK inhibitor

CHK1 and CHK2, which are essential for maintaining DNA integrity, have roles in cell cycle regulation and response to replication pressure^10^. CHK1 is the main effector downstream of ATR and is phosphorylated mainly by ATR on Ser317 and SER345. Activated CHK1 triggers S and G2/M phase checkpoints. CHK1 is critical for cell cycle regulation and response to replication pressure, as well as maintenance of genomic integrity. CHK2 is also a serine/threonine kinase and is the main downstream effector of ATM. CHK2 can repair and modify DSBs in the DDR pathway, regulate S phase and G2/M cell cycle arrest, and induce apoptosis when the DNA is irreparable. By inhibiting the above pathways, CHK inhibitors have a role in regulating the cell cycle and treating OC.

CHK1 and CHK2 also participate in the cell cycle by inhibiting cell division at the S and G2/M checkpoints and allowing time for DNA repair. Tumor cells with TP53 mutation rely on these checkpoints, suggesting that there could be a benefit of CHK inhibition that could prevent progression and tumor growth. The role of the CHK1/2 inhibitor, prexasertib, in HGSOC has been determined in combination with olaparib in a phase I that demonstrated some anti-tumoral activity (4 of 18 BRCA1-mutant, PARPi-resistant patients with HGSOC achieved partial responses) and mediates HR by decreasing RAD51 foci. The safety profile was acceptable and hematologic toxicity was the most frequent^18^.

Prexasertib (LY2603618) is the first selective CHK1/2 inhibitor with efficacy in reducing myocardial toxicity while improving pharmacokinetic characteristics. Prexasertib has shown monotherapy efficacy and increased sensitivity to platinum and olaparib in mouse tumor transplantation models. The completed phase II trial (NCT02203513) enrolled 28 patients with BRCA wild-type HGSOC to evaluate the efficacy of intravenous prexasertib. Cycle E upregulation, CEEN1 amplification, or protein overexpression in 19 of the 24 assessable patients rendered the patients more sensitive to normal cell cycle disruption to recognize and repair DNA damage. Eleven of 19 patients (58%) with platinum-resistant OC benefited from treatment with prexasertib^19^. The promising preclinical studies of ATR/CHK1/WEE1 inhibitors in PARPi-resistant cancers prompted the initiation of early phase clinical trials (**Table 1**).

**Table 1:** Clinical trial(s) targeting cell cycle checkpoints

Trial	Phase	Notable Common Grade AEs (≥10%)	Drugs	Results	Time	Reference
DUETTE NCT04239014	Randomized, double-blinded, placebo-controlled phase II	N/A	Ceralasertib + olaparib	No publications available	2020	DUETTE: a phase II randomized, multicenter study to investigate the efficacy and tolerability of a second maintenance treatment in patients with platinum-sensitive relapsed epithelial ovarian cancer, who have previously received poly(ADP-ribose) polymerase (PARP) inhibitor maintenance treatment

**Figure 1:**
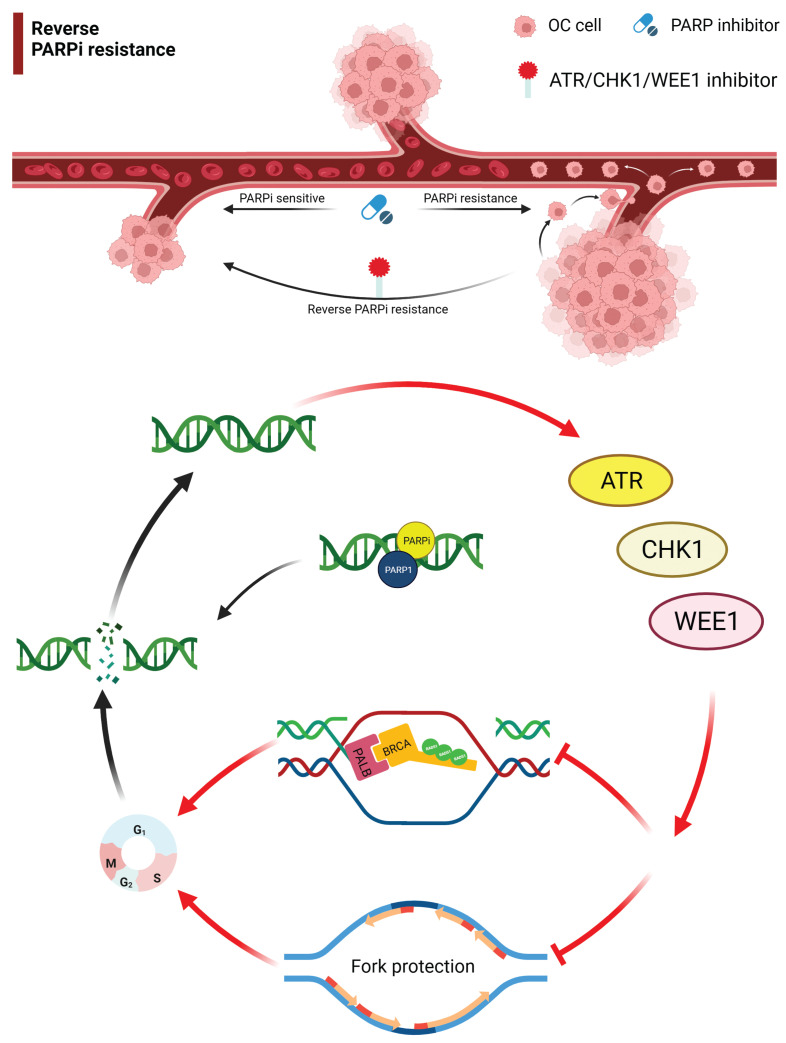
Targeting the ATR/CHK1/WEE1 pathway overcomes PARP inhibitor resistance. Monotherapy or combination therapies with ATR/CHK1/WEE1 inhibitors overcome PARP inhibitor resistance by disrupting both HR and replication fork protection, leading to accumulation of DNA damage throughout the cell cycle and increased cell death. OC, ovarian cancer; ATR, ataxia telangiectasia and Rad3-related; CHK1, cell cycle checkpoint kinase 1.

This article reviewed the mechanism of action for ATM, ATR, WEE1, and CHK1/2 inhibitors and the progress in the treatment of drug-resistant OC. Monotherapy or combination therapy with ATR/CHK1/WEE1 inhibitors overcome PARPi resistance by disrupting homologous recombination and replication fork protection, leading to accumulation of DNA damage throughout the cell cycle and increased cell death (**Figures 1** **and 2**)^20^. The combination of different targeted drug therapies to reduce the development of drug resistance is worthy of further study. A PARPi combined with a DDR-pathway-related inhibitor may be a salvage treatment for OC patients with multiple recurrences and platinum and PARPi resistance. Although the specific underlying mechanism has not been established, good efficacy has been achieved in different clinical studies; however, additional studies are warranted to understand the underlying mechanism in the future.

**Figure 2:**
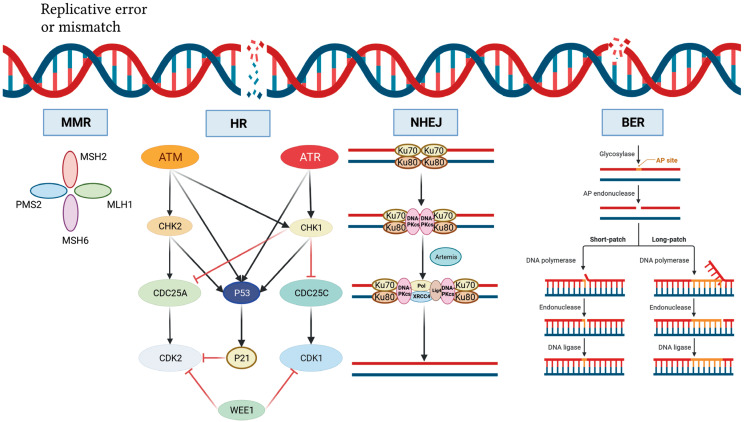
DNA damage repair mechanism. DNA double-strand breaks activate ATM, which phosphorylates and activates CHK2, which in turn phosphorylates and inactivates cdc25A, preventing CHK2 from removing the inactivating phosphate on CDK2, thereby inhibiting S-phase entry and progression. Both ATM and CHK2 phosphorylate p53, resulting in transactivation of p21 to inhibit CDK2. SS-DNA (e.g., at stalled replication forks) activates ATR, which phosphorylates and activates CHK1, which in turn phosphorylates and inactivates cdc25c, thus preventing CHK1 from removing the inactivating phosphate on CDK1 and inhibiting G2/M progression. There is substantial crosstalk between the two pathways with CHK1 also being a target of ATM and cdc25A a target of CHK1 and both ATR and CHK1 targeting p53. In addition, DNA damage activates WEE1, which phosphorylates and inactivates both CDK1 and CDK2. Black arrows indicate main activation pathways, grey arrows are secondary pathways and red lines indicate inhibition. MMR, mismatched repair; HR, homologous recombination; NHEJ, non-homologous end joining; BER, base excision repair.

In conclusion, the evolving landscape of PARPi resistance in OC demands novel therapeutic strategies. Targeting the ATR/ATM/CHK1/WEE1 pathway presents a rational approach to address this challenge. Preclinical studies of cell cycle checkpoint inhibitor monotherapy in combination with PARPi therapy have shown promise, whereas the results of clinical trials must be interpreted with caution. Overlapping toxicities between PARPi therapy and cell cycle checkpoint inhibitors pose a potential challenge. A crucial challenge in practice will be to identify and select patients for the appropriate therapy or combination strategies. However, efforts are needed to decrease overlapping toxicity by modifying the structure of the drug and defining the correct timing of dosing to maximize the therapeutic index.
